# V_2_O_5_-C-SnO_2_ Hybrid Nanobelts as High Performance Anodes for Lithium-ion Batteries

**DOI:** 10.1038/srep33597

**Published:** 2016-09-28

**Authors:** Linfei Zhang, Mingyang Yang, Shengliang Zhang, Zefei Wu, Abbas Amini, Yi Zhang, Dongyong Wang, Shuhan Bao, Zhouguang Lu, Ning Wang, Chun Cheng

**Affiliations:** 1 Department of Materials Science and Engineering and Shenzhen Key Laboratory of Nanoimprint Technology, South University of Science and Technology, Shenzhen 518055, China; 2 Single-Molecule Detection and Imaging Laboratory, Shenzhen Institutes of Advanced Technology, Chinese Academy of Sciences, Shenzhen, 518055, China; 3 Department of Physics, Hong Kong University of Science and Technology, Hong Kong, China; 4 Institute for Infrastructure Engineering, Western Sydney University, Kingswood, NSW 2751, Australia

## Abstract

The superior performance of metal oxide nanocomposites has introduced them as excellent candidates for emerging energy sources, and attracted significant attention in recent years. The drawback of these materials is their inherent structural pulverization which adversely impacts their performance and makes the rational design of stable nanocomposites a great challenge. In this work, functional V_2_O_5_-C-SnO_2_ hybrid nanobelts (VCSNs) with a stable structure are introduced where the ultradispersed SnO_2_ nanocrystals are tightly linked with glucose on the V_2_O_5_ surface. The nanostructured V_2_O_5_ acts as a supporting matrix as well as an active electrode component. Compared with existing carbon-V_2_O_5_ hybrid nanobelts, these hybrid nanobelts exhibit a much higher reversible capacity and architectural stability when used as anode materials for lithium-ion batteries. The superior cyclic performance of VCSNs can be attributed to the synergistic effects of SnO_2_ and V_2_O_5_. However, limited data are available for V_2_O_5_-based anodes in lithium-ion battery design.

As one of the most important energy storage devices, lithium-ion batteries (LIBs) have been extensively studied in recent years owing to their numerous merits such as high energy density, environmental friendliness and light weight^1^,^2^,^3^. Although graphite is a dominant anode material for commercial LIBs, its relatively low theoretical capacity (372 mAhg^−1^) significantly impedes the development of high energy density LIBs^4^,^5^.Transition metal oxides have attracted more interest than commercial graphite as the anode in lithium ion batteries because of their high theoretical capacity and abundant supply of raw materials in nature^6^,^7^,^8^,^9^,^10^,^11^,^12^.

To serve as the anode for LIBs, 2-dimentional nanostructured active materials are ideal choices because of their short Li-ion diffusion distance, facile strain relaxation upon electrochemical cycling, and very large surface area to volume ratio in order to contact well with the electrolyte, all of which improve the capacity and life-cycle of LIBs^13^,^14^,^15^,^16^. In ordinary batteries, however, nanomaterials are often self-aggregated due to the high surface energy. This reduces the effective contact area of active materials, conductive additives, and electrolyte. How to effectively increase the scale of the contact area and take full advantage of nanoscale active materials are still a challenge and of great importance.

V_2_O_5_ is intensively studied materials both as cathode and anode for LIBs because of a high specific capacity, natural abundance, and relatively low cost^17^,^18^. If consider a fully reduction from V^5+^ to V^0^, V_2_O_5_ gains a theoretical capacity of 1472 mAhg^−1^, the highest capacity among all metal oxides, and thus can be an ideal component for high energy anodes^19^,^20^,^21^,^22^. Despite this unique property, there is limited data on the high potential capacity of the V_2_O_5_ anodes that enables a stable cyclic performance^22^,^23^,^24^. For instance, Liu *et al*. reported double-shelled V_2_O_5_-SnO_2_ nanocapsules which exhibit a reversible capacity of 600 mAhg^−1^ at a rate of 250 mAg^−1^ after 50 cycles^16^. Another study on vanadium oxide aerogels showed a high capacity of 1000 mAhg^−1^ at a rate of 118 mAg^−1^ after 30 cycles^22^. In addition, the structural degradation, poor electrochemical kinetics and low electronic conductivity of V_2_O_5_ have seriously impeded its further development. Researchers have proposed ways to overcome this, for instance, Sun *et al*. coated graphene on amorphous V_2_O_5_ via an atomic layer deposition method to enhance its electronic conductivity and electrochemical activity^19^.

SnO_2_ is another extensively studied anode material due to its abundance, safe lithiation potential and high theoretical capacity (782 mAhg^−1^)^25^. Practical applications of SnO_2_ have shown to suffer from its large volume expansion (up to 250%) and agglomeration during the Li-alloying/dealloying process, resulting in pulverization of the electrode and rapid capacity fading^26^. One of the mitigating strategies is to build up heterostructures of SnO_2_ with other materials that can buffer the excessive volume change. Due to the volume variation during the lithiation/delithiation process, V_2_O_5_ can be a prospective candidate for the mechanical support of SnO_2_ in the form of nanocapsules^16^, nanoscaled mixed oxides^27^, and core-shell nanowires^28^. Therefore, the development of 2-dimentional V_2_O_5_/SnO_2_ anode materials (nanobelts or nanosheets) can be an alternative way to effectively improve Lithium storage properties. For example, nanobelts can be cross-stacked to form the densely packed networks, which have a large amount of neighboring void spaces interconnecting to construct numerous pathways for rapid electrolyte diffusion^29^,^30^. Furthermore, the network structure is always providing highly conductive routes for electron transfer so that electric conduction can be greatly facilitated through this way^31^,^32^.

Inspired by previous studies, we develop a simple strategy of coating SnO_2_ on V_2_O_5_ nanobelts by glucose link to achieve high-power-density and high-energy density LIBs. Using a simple two-step hydrothermal method, ultrathin V_2_O_5_-carbon-SnO_2_ hybrid nanobelts (VCSNs) are fabricated with a thickness of approximately 10 nm. Glucose, an excellent linker and carbonating agent, is used to overcome the poor interaction between SnO_2_ and V_2_O_5_. The fabricated LIBs with VCSNs-based anodes exhibited a highly stable cyclic behaviour with a highly reversible capacity of 800 mAhg^−1^ at a current density of 200 mAg^−1^ after 100 cycles. The improved cycling stability and rate capability of these hybrid nanobelts can be attributed to their unique structural design and synergistic effects of SnO_2_ and V_2_O_5_. In addition, the ultrathin feature of VCSNs can improve electron transport, and shorten lithium diffusion paths, leading to an enhanced power density.

## Results

**Figure 1:**
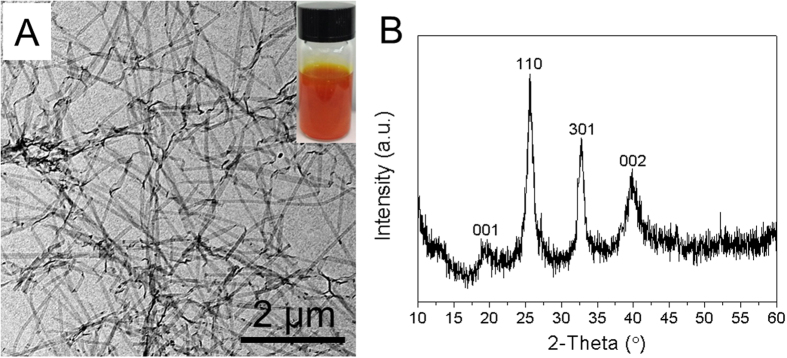
(**A**) TEM image shows the freshly made V_2_O_5_ nanobelt substrates possess widths of 50–80 nm and lengths up to several tens of micrometers with flexible, smooth, thin and almost transparent features. Inset of (**A**) is the ultrathin V_2_O_5_ nanobelts dispersed in water. (**B**) XRD pattern of pure V_2_O_5_ nanobelts.

**Figure 2:**
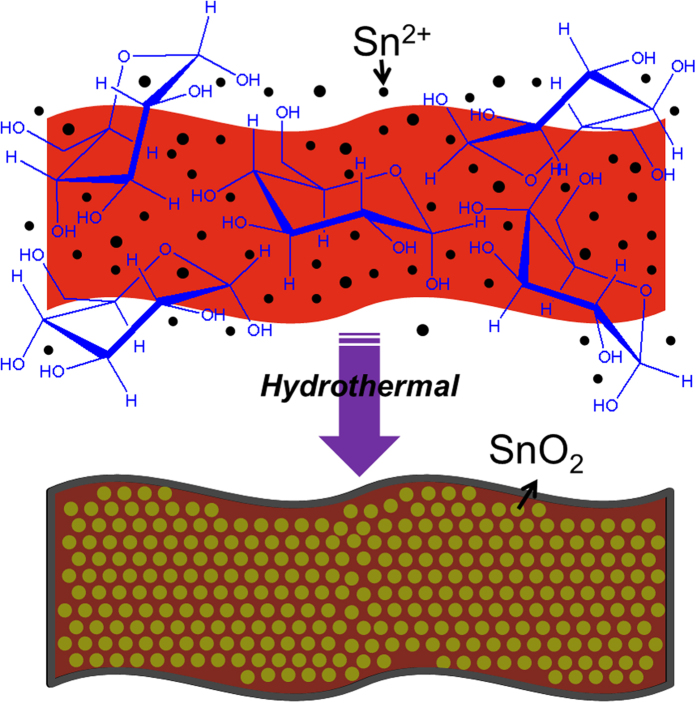
Glucose-induced transformation pathway for the fabrication of the VCSNs.

The ultrathin V_2_O_5_ nanobelts, synthesized using a hydrothermal method, are used as the starting template materials. TEM investigations show that these V_2_O_5_ nanobelts are highly uniform with a thickness of 4 nm and lengths ranging from 800 nm to several micrometres (a large aspect ratio of >200, Fig. 1A). With a high uniformity and relatively large aspect ratios, the V_2_O_5_ nanobelts can serve as excellent templates to support the growth of SnO_2_ nanocrystals through a simple glucose-assisted hydrothermal method. As observed from the XRD pattern in Fig. 1B, the high peaks can be assigned to the orthorhombic V_2_O_5_ (JCPDS No. 40-1296). To grow the nanocrystals, SnCl_2_ is first dissolved in an aqueous solution containing V_2_O_5_ nanobelts. When the hydrothermal temperature is increased, Sn^2+^ hydrolyze to form SnO_2_ crystal nucleus. Then, they are adsorbed onto the surface of V_2_O_5_ nanobelts assisted by a known amount of glucose, fixing the SnO_2_ crystal nucleus on the surface of V_2_O_5_ nanobelts. This association occurs due to the affinity of SnO_2_ and V_2_O_5_ to the –OH groups. As temperature was increased successively to 170 °C, the reaction temperature is higher than the normal glycosidation temperature of glucose, resulting in the carbonization of glucose to form amorphous carbon layer (Fig. 2)^33^,^34^.

**Figure 3:**
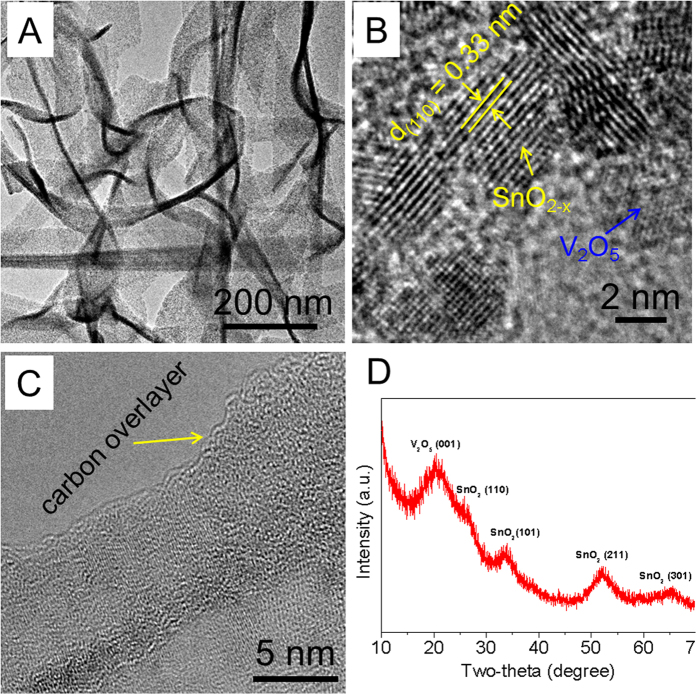
TEM image (**A**–**C**) HRTEM images of the VCSNs. The carbon overlayer is about 2 nm in thickness as indicated in (**C**). (**D**) XRD pattern of V_2_O_5_-based nanocomposites synthesized using the hydrothermal method at 170 °C for 8 h.

Figure 3A shows the TEM image of as-prepared VCSNs. The hybrid nanobelts are characterized as being several micrometres in length and 50~80 nm in width. The collective morphology of hybrid nanobelts displays an excellent uniformity and dispersity as shown in Figure S1 in the Electronic Supplementary Material (ESM). The results of typical HRTEM analyses on these hybrid nanobelts are shown in Fig. 3B,C, demonstrating a dense growth of SnO_2_ nanocrystals on the V_2_O_5_ substrate. The diameter of the anchored SnO_2_ nanocrystals is less than 5 nm. The lattice periodicity of 0.33 nm observed in Fig. 3B corresponds to the spacing of (110) crystal interplanes of tetragonal SnO_2_. The enlarged TEM image recorded at the edge of the nanobelts in Fig. 3C indicates that the entire surface of the nanobelts is covered by a continuous amorphous carbon layer with a thickness of ~2 nm, which comes from the carbonation of glucose. All the XRD peaks of the hybrid nanobelts in Fig. 3D can be well indexed as a tetragonal SnO_2_ phase (JCPDS No. 41-1445) and an orthorhombic V_2_O_5_ phase (JCPDS No. 40-1296). In comparison, the XRD peaks of the VCSNs are relatively broader and weaker than those of the as-synthesized V_2_O_5_ nanobelt template (Fig. 1B). Some peaks are merged in the background; this can be attributed to the smaller size of V_2_O_5_ and SnO_2_ nanocrystals in the composites as identified by the above TEM investigation (Fig. 3B). The EDS pattern (Figure S2 in the ESM) determines that hybrid nanobelts are composed of Sn, V, C, and O, which is consistent with the above TEM and XRD measurements. The surface areas of the as-prepared hybrid nanobelts were investigated using N_2_ sorption isotherm. As indicated in Figure S3, it was found that the hybrid nanobelts have a BET surface area of 132.9 m^2^g^−1^, approximately 4.7 times larger than that of the V_2_O_5_/SnO_2_ sample (28.3 m^2^g^−1^), which prepared no glucose was introduced to the final reaction solution (Figure S4A). This increases the number of surface active sites, which benefits the contact electrode materials and electrolyte, and provides short Li ion pathways.

It was found that glucose plays a critical role in the formation of VCSNs with ideal morphologies (Figure S1). The reaction in the absence of glucose results in irregular and broken nanobelts (Figure S4A). When glucose is replaced with a common nanomaterial synthetic additives, such as polyethylene glycol 2000 (PEG 2000), the resultant nanobelts become small and tangled with lengths ranging from 10 nm to 500 nm (Figures S4B). Therefore, the introduction of glucose not only immobilizes the SnO_2_ crystal seeds on the V_2_O_5_ nanobelt, enabling *in situ* growth of ultradispersed SnO_2_ nanocrystals, but it also benefits V_2_O_5_ nanobelts with their morphology integrity because it functions as an effective physical scaffold.

**Figure 4:**
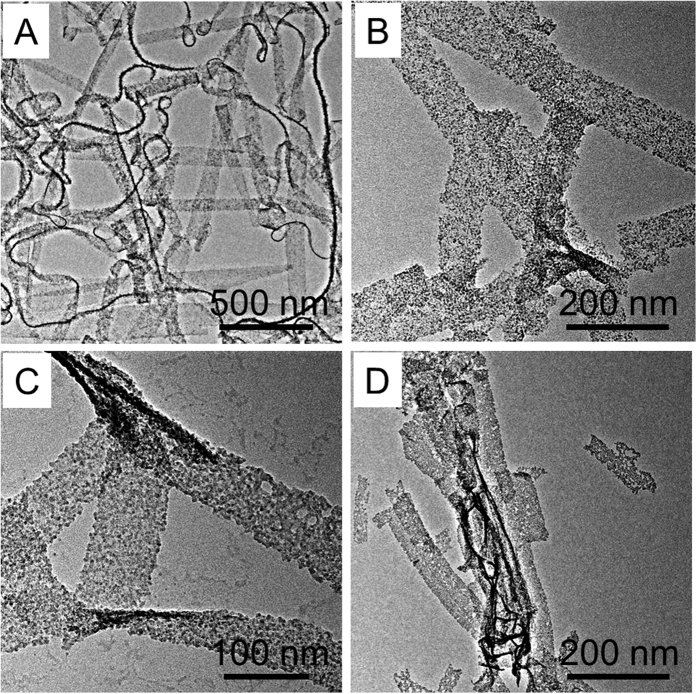
TEM image of the samples synthesized with the addition of different amounts of SnCl_2_. (**A**) 10, (**B**) 60, (**C**) 80, (**D**) 100 mg.

The initial amount of SnCl_2_ significantly affects the final morphologies of hybrid nanobelts. Figure 4 shows the TEM images of the products prepared with the addition of different amounts of SnCl_2_ when other conditions remained unchanged. When the amount of SnCl_2_ is less than 60 mg, hybrid nanobelts with an ideal morphology and high yield are obtained (Fig. 4A,B). When the amount of SnCl_2_ is more than 60 mg, hybrid nanobelts with a pore size of 10–50 nm are formed, and even ruptured in some cases (Fig. 4C,D). The formation of these highly porous hybrid nanobelts can be attributed to the fact that the V_2_O_5_ nanobelts reacted with excessive Sn^2+^ cations through a selective cation exchange. A similar phenomenon has been rationalized for the hydrothermal prepared nanoporous Cd_x_Zn_1-x_S nanosheets based on the cation-exchange reaction^35^.

**Figure 5:**
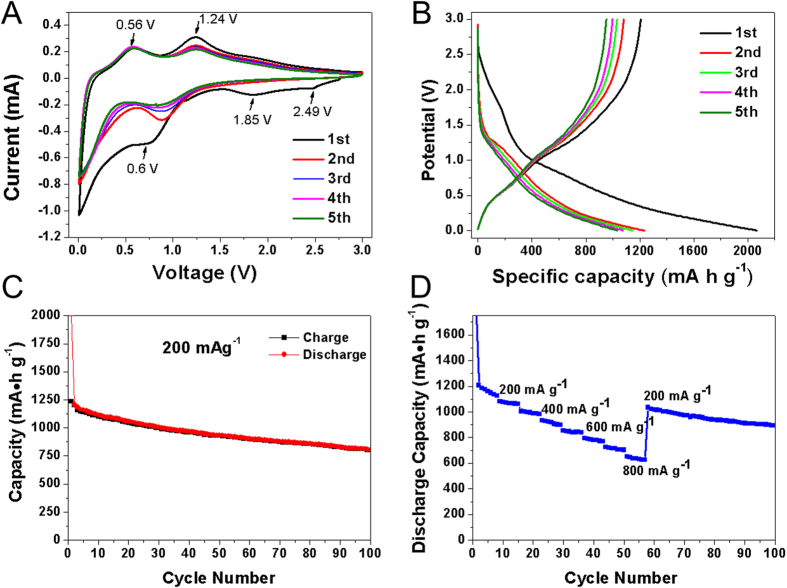
(**A**) Cyclic voltammogram profiles of the VCSNs between 0.01 and 3.0 V at a scan rate of 0.2 mVs^−1^. (**B**) The charge-discharge profiles, and (**C**) cycle performance of the VCSNs based electrode under 200 mAg^−1^, (**D**) Rate performance of the VCSNs at varied current densities. Ag/AgCl used as the reference electrode.

The electrochemical properties of the fabricated VCSNs, as an anode material for LIBs, are presented in Fig. 5. The cell was tested in a two-electrode system coupled with a Li foil as counter electrode. The electrolyte was 1 M LiPF_6_ in a mixture of ethylene carbonate and diethyl carbonate (1:1 by volume). Figure 5A shows the cyclic voltammograms (CV) of the first five consecutive cycles at a scan rate of 0.2 mVs^−1^ within the voltage window of 0.01–3.0 V vs. Li/Li^+^. The pronounced cathodic peaks at 0.6 V in the first cycle can be assigned to the initial reduction of SnO_2_ to Sn (SnO_2_ + 4Li^+^ +  4e^−^ → Sn + 2Li_2_O), the formation of a solid electrolyte interphase layer, and the alloying process to form Li_x_Sn (Sn + xLi^+^ + xe^−^ → Li_x_Sn (0 ≤ x ≤ 4.4)^36^. The reduction peaks located at 2.49 and 1.85 V for the first discharge can be assigned to successive phase transformations upon lithium ion insertion via V_2_O_5_, giving δ-Li_x_V_2_O_5_ and ω-Li_x_V_2_O_5_, respectively^16^. During the first charging process, a strong peak at 0.56 V and a broad peak at 1.23 V correspond to the de-alloying process from Li_x_Sn and partial reversible formation of V_2_O_5_, respectively, which CV behaviour is consistent with those reported in the literature^37^,^38^,^39^, suggesting that they share the same electrochemical reaction pathway. Figure 5B depicts the charge-discharge curves of a Li/V_2_O_5_-carbon-SnO_2_ cell. The first discharge and charge capacities are respectively 2075 and 1205 mAhg^−1^ at a current density of 200 mAg^−1^. During the initial cycle, a large irreversible capacity emerges with an initial coulombic efficiency of 58%. This phenomenon can be attributed to the formation of a solid electrolyte interphase layer on the VCSNs electrode surface. Due to different redox reactions associated with Li insertion/extraction, multiple voltage plateaus can be observed in the first charge and discharge curves (Fig. 5A,B). Although SnO_2_ theoretically possesses a capacity of 782 mAhg^−1 ^25, the lion’s share of the capacity for these VCSNs is from V_2_O_5_ because only 10 wt% of the composite is SnO_2_. When the composite is discharged to 0 V versus Li/Li^+^, if consider that V_2_O_5_ is fully reduced to metallic V, the theoretical capacity of the composite can reach closely to 1404 mAhg^−1^. However, V_2_O_5_ is hardly fully reduced^22^. XPS results (Figure S5 in the ESM) show that when the cell is fully discharged to 0.01 V, the peak of V 2p_3/2_ shifts from the binding energy of 516.9 eV (corresponding to V^5+^, Figure S5A) to lower binding energy of 513.9 eV (corresponding to V^2+^, Figure S5B) with a quite low intensity. Similar results were also reported from previous work^22^. Considering the improbability of a full conversion to V^0^, so the majority of vanadium in the V^2+^ state. The XRD pattern of the pure V_2_O_5_ nanobelts and VCSNs obtained after fully diacharge to 0.01 V is shown in Figure S5C,D. The (111), (200) peaks of VO are observed and the reflection peak can be readily indexed as a cubic VO (JCPDS card No. 65-4054). The (200), (101) peaks of Sn (JCPDS card No. 65-0296, tetragonal) are also observed in Figure S5D. However, the phase of metallic V was not observed in Figure S5C,D, indicating the formation of VO at the end of the discharge step would explain the +2 oxidation state observed in the XPS results. Our estimation of capacity (1404 mAhg^−1^) is close to the measured capacity (1204 mAhg^−1^) of the nanocomposite as an anode material.

Figure 5C shows the cyclic performance of this anode material composite at a current density of 200 mAg^−1^ for 100 cycles. After 50 cycles, the hybrid nanobelts still display a high reversible capacity of 930 mAhg^−1^, and the capacity retention is as high as 84.5% from the 10^th^ cycle. After 100 cycles, the nanocomposites retain a reversible capacity of 800 mAhg^−1^, which demonstrates the outstanding cyclic stability of VCSNs. Two other tests carried out from the same batch. The nanocomposites retained a reversible capacity of 754 mAhg^−1^and 786 mAhg^−1^ after 100 cycles, respectively (Figure S6). In comparison, the cyclic performances of the V_2_O_5_/SnO_2_ composites without a carbon layer and carbon-V_2_O_5_ core-shell nanobelts are provided in Figures S7 and S8, respectively. Under identical testing conditions, after 50 cycles at 200 mAg^−1^, a much faster fading of the capacities occurs and reaches ~518 mAhg^−1^ for V_2_O_5_/SnO_2_ composites and ~411 mAhg^−1^ for carbon-V_2_O_5_ core-shell nanobelts. From the EIS plots (Figure S9), the VCSNs exhibit a much lower resistance than the V_2_O_5_/SnO_2_ composites, as evidenced by the significant reduction in the diameter of the semicircle in the high-frequency region. The lower contact and charge transfer impedances facilitate the Li^+^ ion diffusion and electron transfer which in turn enhance the electrochemical performance of VCSNs.

To evaluate the rate capability, the VCSNs were cycled at various current densities ranging from 100 to 800 mAg^−1^ over a voltage window of 0.01–3.0 V (Fig. 5D). The VCSNs experienced only a small decrease in capacity as the current density increased. For example, at a high current density of 800 mAg^−1^, the VCSNs could still deliver a reversible capacity of about 620 mAhg^−1^. Remarkably, when the current rate was reduced back to 200 mAg^−1^ after 60 cycles, a reversible capacity of about 1005 mAhg^−1^ was retained, which demonstrates the superior rate capability of VCSNs.

## Discussion

**Figure 6:**
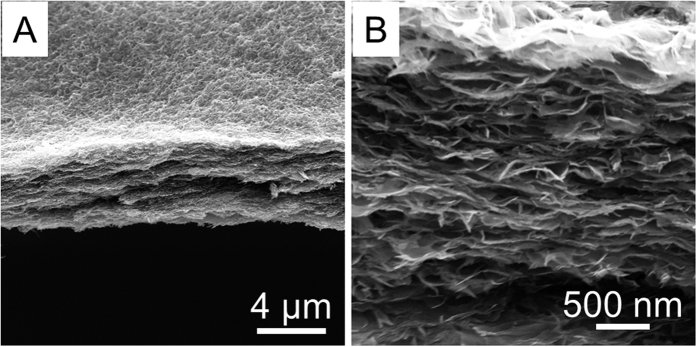
(**A**) Side view of the VCSNs membrane, and (**B**) the status of stacked membranes for the demonstration of the sample’s morphology and texture.

**Figure 7:**
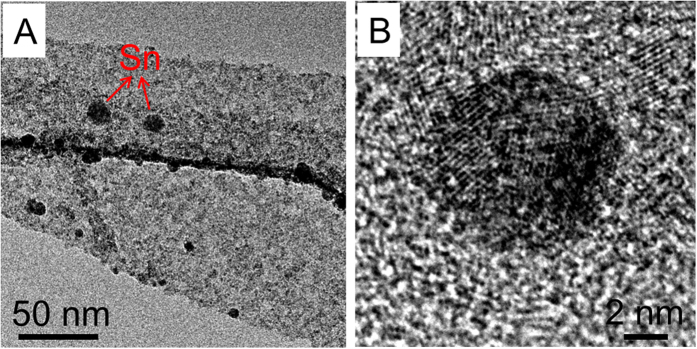
(**A,B**) TEM images of the VCSNs based electrode after full-discharge under 200 mAg^−1^.

The improved cycling stability and rate capability of these hybrid nanobelts can be attributed to the unique design of the nanostructured compositions. Firstly, the ultrathin nanobelt subunits have a short distance for efficient Li^+^ ions diffusion and large electrode-electrolyte contact area for high Li^+^ ions flux across the interface. This leads to an enhanced rate capability^39^,^40^. Secondly, it has been reported that the morphologies and structures of anodes made of pure V_2_O_5_ nanomaterials or their nanocomposites tend to collapse due to the frequent insert/release process of Li^+^ ions which results in a serious degradation of cycling stability^41^. Finally, These hybrid nanobelts are cross-stacked to form densely packed networks. Inside the networks (Fig. 6A,B), it is observed that the membrane has been densely “woven”, and features uniform thickness and evenly distributed nanobelts, both of which provide a solid foundation for the membrane’s following applications in some integrated devices. A large amount of neighboring void spaces are interconnected to construct numerous pathways for rapid electrolyte diffusion, which is also the rate-limiting step to determine the LIB’s rate capability. Moreover, in the present case, the soft carbon layer acts as an excellect physical scaffold where the ultrathin nanobelt subunits are tightly linked to or embedded in. This effectively counteracts the morphological and structural pulverization of the V_2_O_5_-based nanocomposites. Therefore, the capacity retention of these VCSNs is significantly improved compared with many other V_2_O_5_-based nanostructures^42^,^43^,^44^,^45^. In addition, Sn nanoparticles (generated during the reduction process of SnO_2_ when the nanocomposite is used as an anode) are embedded in the V_2_O_5_ matrix and form an ultrafine metal-oxide electrode (Fig. 7A,B), which is consistent with the above XRD measurements (Figure S5D). The electrode materials made by this process may have some outstanding advantages, such as good tolerance for cyclic volume variations, and high electronic and ionic conductivity^46^.

## Conclusions

Ultrathin V_2_O_5_-carbon-SnO_2_ hybrid nanobelts with a high yield were fabricated using a solution-based method. These nanostructures provide short Li ion diffusion pathways and a high electronic and ionic conductivity supported by a stable structure. By using glucose as a connection linker and carbonation agent for the formation of monodispersed SnO_2_ nanocrystals on V_2_O_5_ nanobelt surfaces, structural pulverization was retarded. As anode materials for LIBs, these hybrid nanobelts exhibit a very high reversible capacity, excellent cyclic performance, and good rate capability. The introduced strategy to control the growth of multicomponent metal oxide could inspire a new way of tailoring nanostructures for the rational design of functional nanocomposites with improved performance for solar light conversion devices, energy storage, and water splitting facilities.

## Methods

### Growth of V_2_O_5_ Nanobelts and VCSNs

All chemicals are of analytical grades and used without further purification. First, ultrathin V_2_O_5_ nanobelts were synthesized from V_2_O_5_ powder using the modified Zhu’s method^47^. Briefly, 0.36 g of V_2_O_5_ powder, 5 mL of 30% H_2_O_2_, and 30 mL of deionized water were mixed until a clear solution was obtained; then 35 mL of this mixture was placed in a 100 mL Teflon autoclave and maintained at 190 °C for 20 hours to generate V_2_O_5_ nanobelts. The resultant brick red floccules were collected using the centrifugation method at 8,000 rpm for 5 min, and subsequently washed using pure ethanol three times. Finally, the resultant V_2_O_5_ nanobelts were dispersed in 140 mL of deionized water for further use.

VCSNs were fabricated via a simple hydrothermal process. In a typical synthesis, 0.04 g SnCl_2_·2H_2_O was dissolved in a 30 ml solution of V_2_O_5_ nanobelts, then 40 mL of 0.05 M aqueous glucose solution was added while stirring. After 30 minutes, a brown suspension appeared and was transferred to a 100 ml Teflon-lined autoclave, sealed and heated in an oven at 170 °C for 8 hours, and cooled to room temperature naturally. The resultant black product was collected through centrifugation at 6,000 rpm for 5 min, then washed at least four times by distilled water and pure ethanol in sequence to remove ions and possible remnants. It was finally dried under a vacuum at 80 °C for 6 hours. The V_2_O_5_/SnO_2_ hybrids were also prepared for comparison and a similar fabrication process was used for the above VCSNs synthesis except that no glucose was introduced to the final reaction solution.

### Sample Characterizations

X-ray diffraction (XRD) patterns were conducted using a Bruker D8 Advanced X-Ray Diffractometer with Ni filtered Cu K-alpha radiation (λ = 1.5406 Å) at a voltage of 40 kV and a current of 25 mA. Transmission electron microscope (TEM) images and high-resolution transmission electron microscopic (HRTEM) images were captured and energy dispersive X-ray spectroscopy (EDS) analysis was conducted using a JEOL-2010 microscope at an accelerating voltage of 200 kV. Nitrogen adsorption measurements were taken at 77 K using a Micromeritics ASAP 2020 system utilized for Barrett-Emmett-Teller (BET) calculations for surface area. The nitrogen sorption measurement was performed on Autosorb-6B at a temperature where N_2_ remains in a liquid state (−196 °C).

### Electrochemical measurements

Electrochemical tests were carried out in 2032 coin-type cells. The working electrodes consisted of 80 wt% of the active material (VCSNS), 10 wt% of conductive carbon black (Super-P-Li), and 10 wt% of polymer binder (polyvinylidene fluoride, PVDF) was fabricated by casting a slurry onto a copper foil (99.6%, Goodfellow). The amount of the active material for the electrochemical test was 1.24 mg. The electrolyte was 1 M LiPF_6_ in a mixture of ethylene carbonate and diethyl carbonate (1:1 by volume). Lithium foils were used as the counter electrode separated from the working electrode by glass fibres. Cell assembly was conducted in an Ar-filled glovebox with moisture and oxygen concentration below 1.0 ppm. Charge-discharge tests were performed on a NEWARE battery tester. For anode performance measurements, the cells were charged/discharged in a voltage window of 0.01–3.0 V at different current densities. Cyclic voltammogram (CV) measurements were performed on a CH Instrument model 600C electrochemical workstation at a scan rate of 0.2 mVs^−1^. Electrochemical impedance spectroscopy (EIS) measurements were conducted for the working electrode in the frequency range of 100 kHz to 0.01 Hz with ac perturbation of 5 mV. The EIS data were analyzed using Nyquist plots, with both the imaginary part (Z′) and real part (Z″) of impedance considered.

## Additional Information

**How to cite this article**: Zhang, L. *et al*. V_2_O_5_-C-SnO_2_ Hybrid Nanobelts as High Performance Anodes for Lithium-ion Batteries. *Sci. Rep.* **6**, 33597; doi: 10.1038/srep33597 (2016).

## Supplementary Material

Supplementary Information
