# Supplementary material for: V2O5-C-SnO2 Hybrid Nanobelts as High Performance Anodes for Lithium-ion Batteries
Source: Sci Rep. 2016 Sep 28;6:33597. doi: 10.1038/srep33597 (PMC5039413; doi:10.1038/srep33597)
Supplement: Supplementary Information [file srep33597-s1.doc]

Supplementary Information

**V_2_O_5_-C-SnO_2_ Hybrid Nanobelts as High Performance Anodes for Lithium-ion Batteries**

Linfei Zhang ^1, 4#^, Mingyang Yang^1#^, Shengliang Zhang^1^, Zefei Wu^2^, Abbas Amini^3^, Yi Zhang^1^, Dongyong Wang^1^, Shuhan Bao^1^, Zhouguang Lu^1^, Ning Wang^2^, Chun Cheng^1*^

1. Department of Materials Science and Engineering and Shenzhen Key Laboratory of Nanoimprint Technology, South University of Science and Technology, Shenzhen 518055, China

2. Department of Physics, Hong Kong University of Science and Technology, Hong Kong, China

3. Institute for Infrastructure Engineering, Western Sydney University, Kingswood, NSW 2751, Australia

4. Single-Molecule Detection and Imaging Laboratory, Shenzhen Institutes of Advanced Technology, Chinese Academy of Sciences, Shenzhen, 518055, China


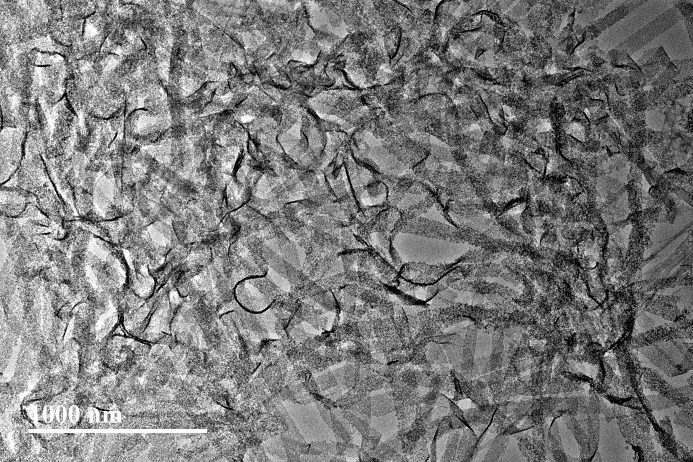


**Figure S1.** Low-magnification TEM image of the VCSNs synthesized in the presence of glucose.


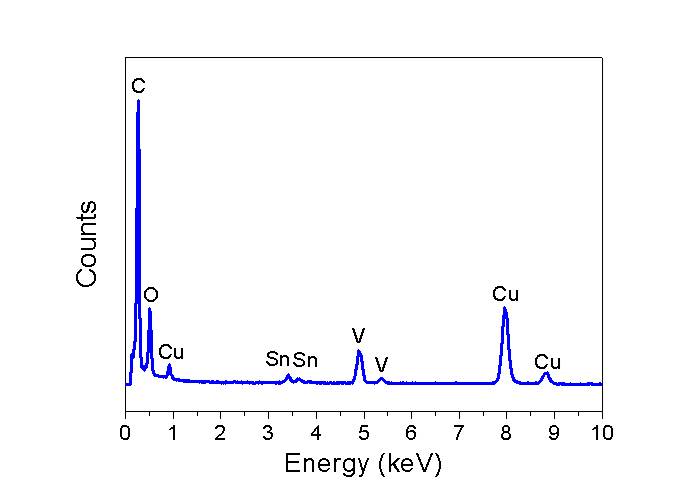


**Figure S2.** EDX spectrum shows the elemental composition of the VCSNs. The weight ratio of V and Sn is 10. The EDS analysis confirms that the representative peaks corresponding to Sn and O elements exist and the Sn/O atomic ratio is about 30:66.


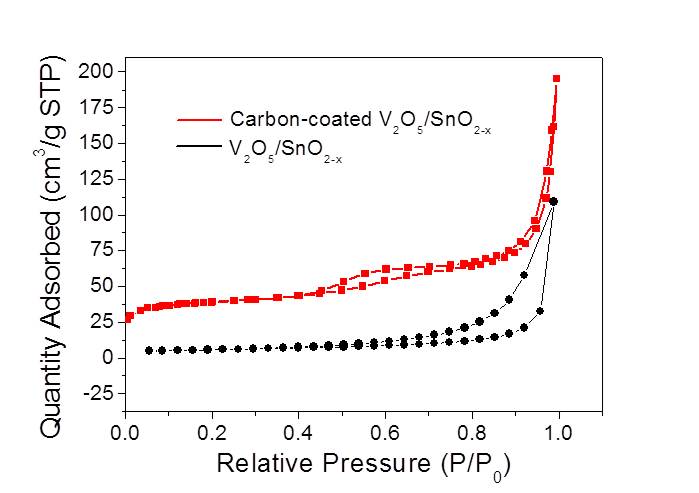


**Figure S3.** Nitrogen adsorption/desorption isotherms of the VCSNs, and the V_2_O_5_/SnO_2_ samples, showing that the hybrid architecture has a higher surface area of 132.9 m^2^/g.


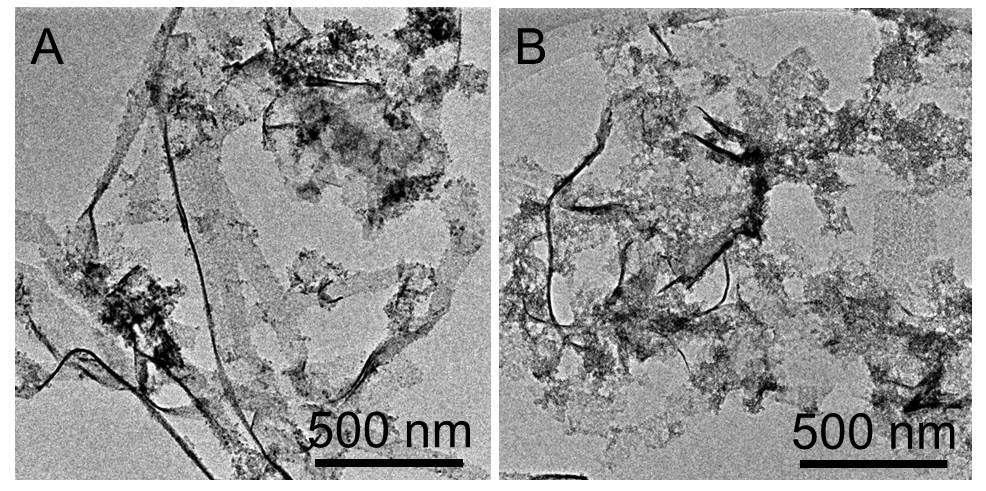


**Figure S4.** TEM images of V_2_O_5_/SnO_2_ nanocomposites synthesized without glucose (A) and with PEG 2000 (B).


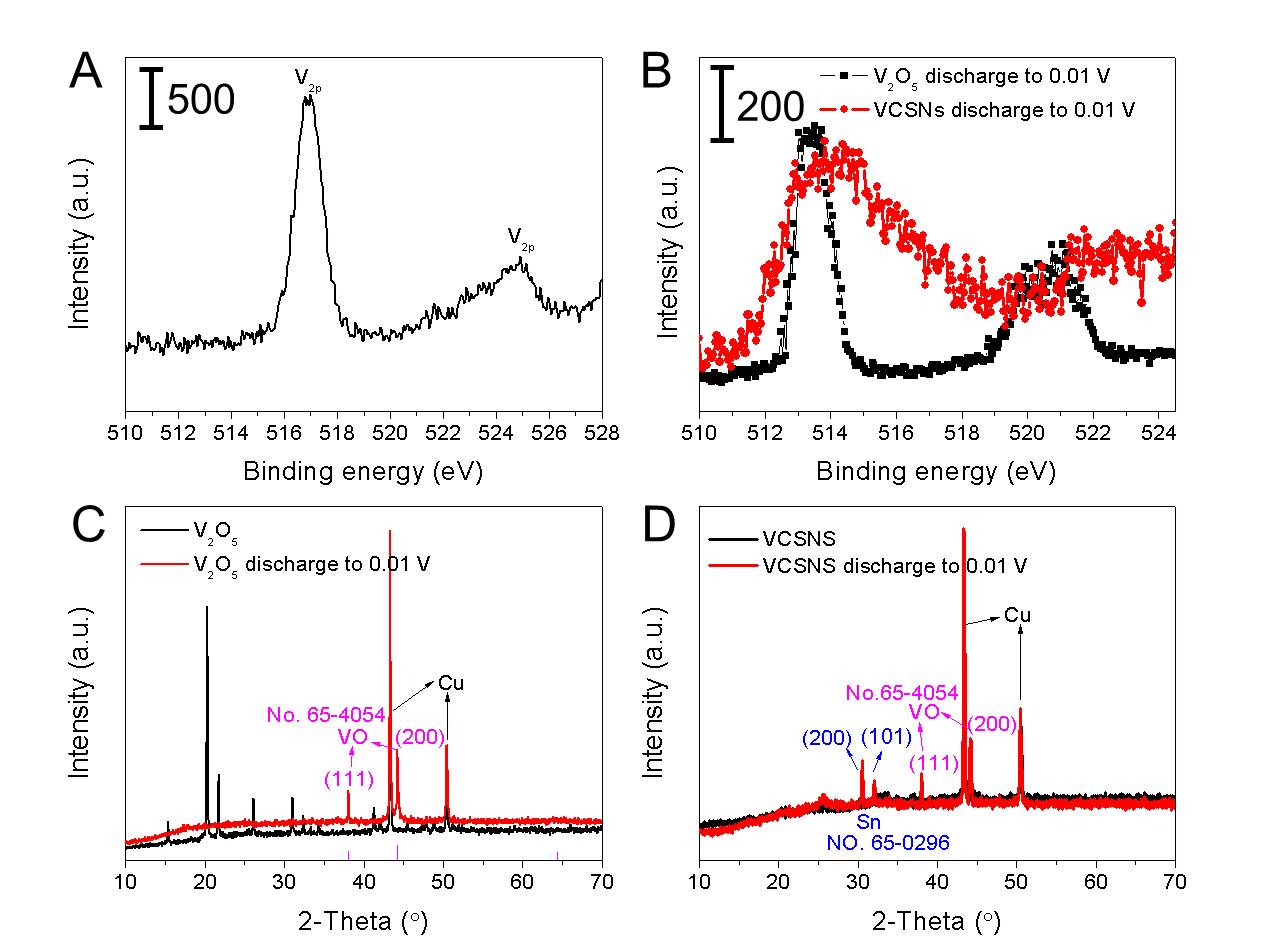


**Figure S5.** XPS spectra of fully charge (A) of the VCSNs, and fully discharge (B) of the V_2_O_5_ and VCSNs, and XRD pattern of pure V_2_O_5_ nanobelts (C) and VCSNs (D) fully discharge to 0.01 V.


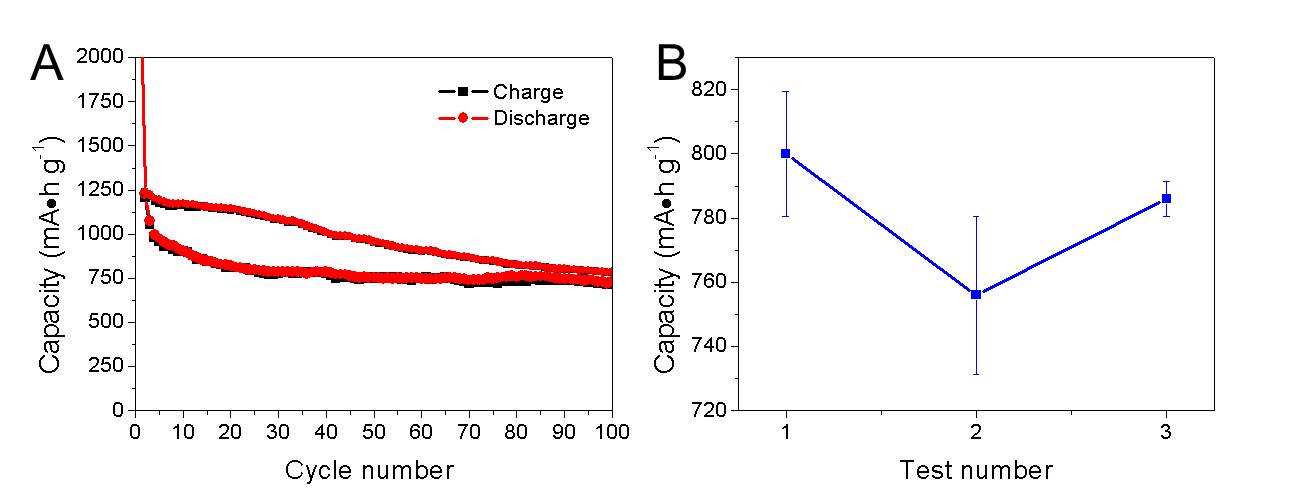


**Figure S6.** (A) Cycle performance of the VCSNs based electrode under 200 mAg^-1^ for 100 cycles (another 2 times). (B) The error bars were obtained with at 3 repeated trials.

**
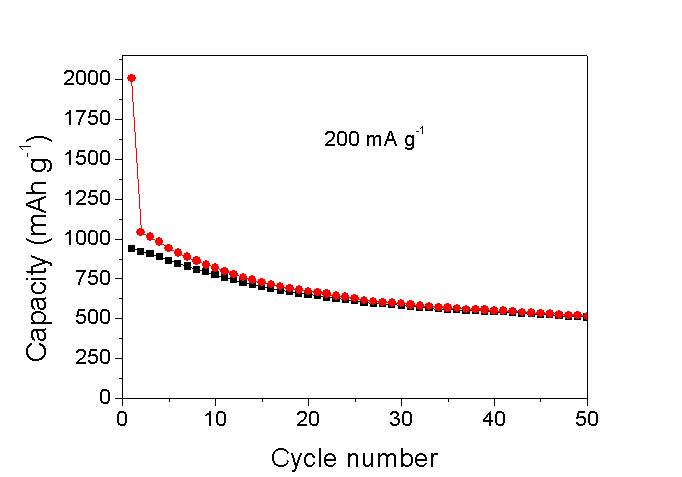
**

**Figure S7.** Charge and discharge capacities versus cycle number of V_2_O_5_/SnO_2_ nanocomposites at the current density of 200 mAg^-1^.


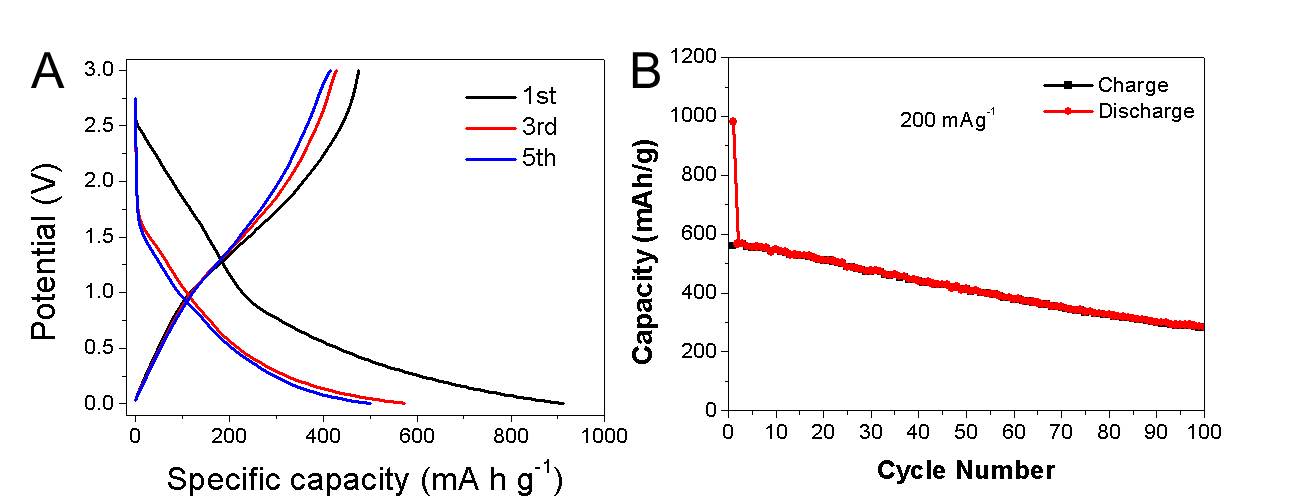


**Figure S8.** (A) The discharge−charge voltage profiles and (B) the cycling performance of the carbon-V_2_O_5_ core-shell nanobelts over the voltage range of 0.01−3.0 V vs. Li/Li^+^ at the current density of 200 mAg^-1^.

**
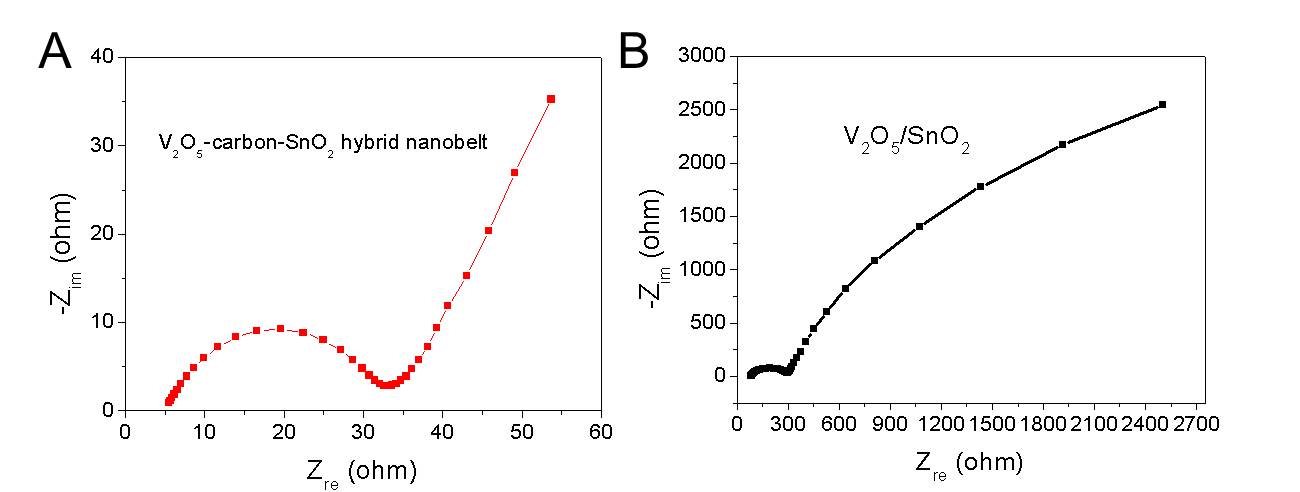
**

**Figure S9.** Nyquist plots of the electrodes composed of the VCSNs and V_2_O_5_/SnO_2_ composites.
